# Body weight and premature retirement: population-based evidence from Finland

**DOI:** 10.1093/eurpub/ckab116

**Published:** 2021-07-19

**Authors:** Jutta Viinikainen, Santtu Tikka, Mikko Laaksonen, Tuija Jääskeläinen, Petri Böckerman, Juha Karvanen

**Affiliations:** 1 Jyväskylä University School of Business and Economics, University of Jyväskylä, Jyväskylä, Finland; 2 Department of Mathematics and Statistics, University of Jyväskylä, Jyväskylä, Finland; 3 The Finnish Centre for Pensions, Helsinki, Finland; 4 Finnish Institute for Health and Welfare, Helsinki, Finland; 5 Labour Institute for Economic Research, Helsinki, Finland; 6 IZA Institute of Labor Economics, Bonn, Germany

## Abstract

**Background:**

Health status is a principal determinant of labour market participation. In this study, we examined whether excess weight is associated with withdrawal from the labour market owing to premature retirement.

**Methods:**

The analyses were based on nationally representative data from Finland over the period 2001–15 (*N* ∼ 2500). The longitudinal data included objective measures of body weight (i.e. body mass index and waist circumference) linked to register-based information on actual retirement age. The association between the body weight measures and premature retirement was modelled using cubic b-splines via logistic regression. The models accounted for other possible risk factors and potential confounders, such as smoking and education.

**Results:**

Excess weight was associated with an increased risk of premature retirement for both men and women. A closer examination revealed that the probability of retirement varied across the weight distribution and the results differed between sexes and weight measures.

**Conclusion:**

Body weight outside a recommended range elevates the risk of premature retirement.

## Introduction

Populations in industrialized countries are aging rapidly.^1^ This structural change increases the dependency ratio and puts significant pressure on public finances. As a policy proposal to mitigate financial sustainability concerns, countries have striven to find ways to extend people’s working lives. To accomplish this goal, many countries have increased the minimum retirement age. However, at the same time, premature retirement (i.e. retirement before the expected age) is widespread. This reduces the effective retirement age and dilutes efforts to increase labour supply in older age groups.^2^

Health status is an important determinant of labour market participation.^3^^,^^4^ The physical health of older people has considerably improved during the past few decades. However, this positive development is threatened by the increasing prevalence of obesity, which occurs in almost all industrialized countries,^5^ including Finland.^6^ Obesity is closely linked to severe chronic health conditions^7^ that may substantially weaken working capacity and lead to early withdrawal from the labour market.

Obesity may affect labour market participation not only by causing chronic health conditions but also by hindering the capability to work, especially in jobs that require physical exertion. Studies have found that excess body weight is associated with exit from the labour market, especially through disability retirement.^8–10^ Swedish studies based on male military conscripts have identified a J-shaped association between body mass index (BMI) and disability retirement.^11^^,^^12^ J-shaped association refers to the situation where the risk of premature retirement rapidly decreases with increasing BMI up to a certain threshold, after which the risk steadily increases with BMI. Similar findings have been made in some other studies.^9^^,^^13^ Meanwhile, most studies that examined the association between BMI and premature retirement relied solely on self-reported information. Moreover, BMI does not account for differences in body composition. Central obesity is regarded as the most harmful form of obesity since visceral fat is metabolically more active than subcutaneous fat. Thus, evidence on premature retirement using obesity indicators other than BMI is limited.^14^^,^^15^

In this article, we examine how body weight is associated with premature retirement by using nationally representative register-based information on actual retirement age, which is linked to objective measures of body weight. We contribute to the literature in three ways: First, instead of using a single measure for body weight, we used both BMI and waist circumference (WC), a measure of abdominal obesity, in the analyses. Second, our estimation model allowed body weight measures and premature retirement to have a flexible non-linear relationship. Third, we considered whether sex, socioeconomic status, smoking habits, marital status and frequent hospital visits (as an indicator of poor health status) explain the relationship between body weight and premature retirement.

## Methods

### Study sample

We used nationally representative Finnish data on prime working-age individuals who were between 30 and 47 years of age at the beginning of the study period (2001–15). The data were combined from three sources. Information on BMI, WC and education originated from the Health 2000 population data.^16^ These data were linked to two administrative registers. The first register is data from the Finnish Centre for Pensions, which include information on actual retirement ages. The second register is data from the Care Register for Health Care, which contain information on hospital patient discharges. The data were linked using personal identity codes. The ethics committee for Research in Epidemiology and Public Health approved the data collection and register linkages for the Health 2000 Survey. The participants were fully informed; they participated in the surveys voluntarily and provided written informed consent for the use of their data. Permissions for the use of the register-based data were obtained from the data controllers.

The Health 2000 Survey was conducted in 2000–01 when a total of 9922 Finns who were at least 18 years old were invited to participate in the study. The participation rate in the health examinations was 84%.^16^ To account for non-participation, we used survey weights that have been calibrated to the data.^17^ The register-based data drawn from the Finnish Centre for Pensions cover all pension recipients in the earnings-related pension system. Individuals with no employment history receive national pension only and are not included in this register. Furthermore, information on hospitalizations was obtained from the Care Register for Health Care, which are nationwide data containing information on patients discharged from hospitals. The completeness and accuracy of the data have been shown to be excellent.^18^

Supplementary appendix figure A1 depicts a flowchart of the construction of the study sample. It shows the various data sources and exclusion criteria of individuals at each stage. In this study, we focused only on the first retirement period between 2001 and 2015. We excluded persons who had retired before 2001 and who were 48 years of age or above in 2000. This age restriction enabled us to focus on individuals who can potentially retire prematurely during the entire follow-up period. Table 1 documents the descriptive statistics of the study sample by sex and Supplementary appendixtable A1 by BMI category.

**Figure 1:**
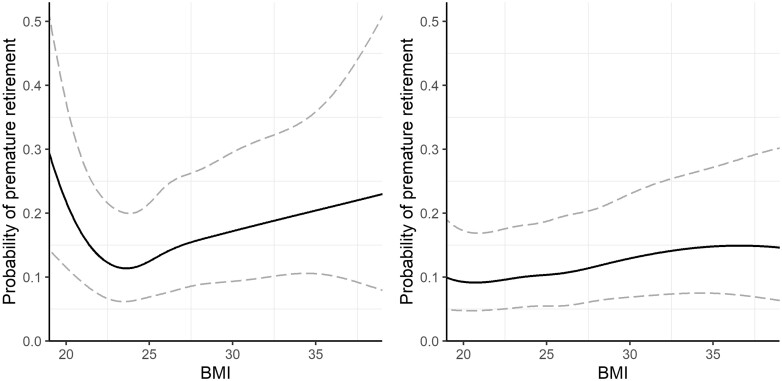
The relationship between BMI and the probability of premature retirement was modelled by b-splines with six degrees of freedom. For the predictions, the background variables were set as follows: 39 years of age, high density area of residence, HYKS university hospital district, primary level of education, zero hospital visits during the past 5 years, never smoked, not married or cohabiting. Note: *N* = 1176 (men), 1336 (women)

**Table 1:** Descriptive statistics of the study sample

	Men (*N* = 1182)	Women (*N* = 1345)	Total (*N* = 2527)
Age	38.6 (5.1)	38.4 (5.0)	38.4 (5.0)
BMI	26.4 (4.0) [0]	25.3 (4.9) [2]	25.8 (4.5) [2]
WC	94.8 (10.8) [9]	83.8 (12.5) [20]	88.9 (13.0) [29]
Smoking	[6]	[6]	[12]
Never	669 (56.9%)	914 (68.3%)	1583 (62.9%)
Occasionally	114 (9.7%)	110 (8.2%)	224 (8.9%)
Daily	393 (33.4%)	315 (23.5%)	708 (28.2%)
Education	[6]	[6]	[12]
Lowest	220 (18.7%)	202 (15.1%)	422 (16.8%)
Middle	583 (49.6%)	478 (35.7%)	1061 (42.2%)
Highest	373 (31.7%)	659 (49.2%)	1032 (41.0%)
Area	[0]	[0]	[0]
HYKS	393 (33.2%)	445 (33.1%)	838 (33.2%)
TYKS	163 (13.8%)	183 (13.6%)	346 (13.7%)
TaYS	258 (21.8%)	290 (21.6%)	548 (21.7%)
KYS	189 (16.0%)	230 (17.1%)	419 (16.6%)
OYS	179 (15.1%)	197 (14.6%)	376 (14.9%)
Region	[0]	[0]	[0]
High-density urban	746 (63.1%)	876 (65.1%)	1622 (64.2%)
Low-density urban	173 (14.6%)	153 (11.4%)	326 (12.9%)
Rural	263 (22.3%)	316 (23.5%)	579 (22.9%)
Marital status	[6]	[5]	[11]
Married or cohabiting	318 (27.0%)	308 (23.0%)	626 (24.9%)
Other	858 (73.0%)	1032 (77.0%)	1890 (75.1%)
Hospital visits	[0]	[0]	[0]
0 visits	842 (71.2%)	732 (54.4%)	1574 (62.3%)
1–3 visits	257 (21.7%)	416 (30.9%)	673 (26.6%)
>3 visits	83 (7.0%)	197 (14.6%)	280 (11.1%)

Note: For continuous variables, the mean, standard deviation and the number of missing observations are denoted as Mean, (SD) and [NA], respectively. For categorical variables, the counts and proportions are indicated as N (%), whereas the number of missing observations is indicated as [NA].

### Measurements

In Finland, the retirement age is based on legislation and agreements between the central organizations of employees and employers. Before the pension reform in 2005, the normal retirement age in the private sector was 65 years. However, many state and municipal employees and some special occupations had a lower retirement age. The pension reform in 2005 made old-age retirement flexible between the ages of 63 and 68. Nowadays and among the cohorts in this study, disability retirement is by far the most common reason for premature retirement.^19^ A small proportion (20%) of those who retired prematurely in our sample retired via a non-disability-related pension. We excluded this small subset of individuals and focused only on exits from the labour market via disability-related pension before the normal retirement age (i.e. before 63 years of age). The outcome variable is one for those who retired or died before the age of 63 over the period of 2001–15 and zero otherwise.

All anthropometric measures originated from the Health 2000 Survey and were based on measurements conducted by trained nurses. Height was measured using a tape measure fastened to the wall, and weight was measured with a spring scale (Biospace, Inbody 3.0). After inserting the height into the scale, the machine automatically calculated the BMI (kg/m^2^).^20^ We also used WC as an alternative weight measure to BMI to predict premature retirement. WC was measured on the bare skin in a standing position from the mid-point between the lowest rib bones and the high point of the iliac crest.

Furthermore, information on baseline characteristics (sex, age, area of residence, education and marital status) were collected from questionnaires and interviews in the Health 2000 Survey. Moreover, information on the highest level of education completed was reported using three categories: primary, secondary and tertiary education. Additionally, information on the geographical area of residence was based on five university hospital districts (HYKS = Helsinki, TYKS = Turku, TaYS = Tampere, KYS = Kuopio, OYS = Oulu). We further characterized the region of residence using three indicators of population density (high density urban, urban, rural area) based on the official classification by Statistics Finland. Marital status was represented with an indicator variable, which equalled one if the respondent was married or cohabiting and zero otherwise. Smoking status was obtained using a 3-point scale question in the Health 2000 Survey: ‘Do you currently smoke?’ (no; yes, daily; yes, occasionally). Based on these answers, three smoking habit indicators were constructed. The number of hospital visits during 1995–2000 originated from the Care Register for Health Care and was measured with three categories: 0 visits, 1–3 visits and more than 3 visits.

### Statistical analyses

We used logistic regression to model the association between BMI and premature retirement (Model 1) as well as WC and premature retirement (Model 2). The analyses were performed using R and the ‘survey’ package, which takes the survey design into account and applies the inverse probability weights that were used to correct for non-participation and to provide nationally representative results. The models were estimated separately for both sexes.

To account for a potential non-linear relationship between BMI and the probability of premature retirement, we used cubic b-splines with six degrees of freedom. The splines were constructed separately for both sexes by placing knots at the lower quartile, median and upper quartile of the empirical BMI distributions of the sexes. As a sensitivity check, we also constructed the splines with alternative degrees of freedom, and we found that having more than six degrees of freedom did not meaningfully improve the fit. Moreover, using fewer than six degrees of freedom resulted in splines that fit poorly. We also experimented with alternative knot placements but did not observe any meaningful changes in the model fit or results. The b-splines for WC were constructed in the same manner.

We controlled for age, age squared, area of residence (geographical location and population density), education and marital status in all the models. Smoking has been linked to poor health and a higher risk of early retirement (e.g. Bengtsson and Nilsson^21^). Therefore, we also controlled for smoking habits in all the models. Furthermore, poor health has been identified as a major predictor of early retirement.^4^ Because pre-existing health conditions may contribute to a change in BMI, we controlled for the number of hospital visits in the baseline model. Since hospitalizations may also be considered as mediators between body mass and premature retirement, we estimated the model without this covariate (Models 3 and 4) in a robustness analysis. As the second robustness test, we investigated whether the relationship between BMI and premature retirement varied with age by fitting a model in which the spline was replaced with a categorical covariate for BMI [underweight (BMI < 18.5), normal weight (18.5 ≤ BMI < 25), overweight (25 ≤ BMI < 30) and obese (BMI ≥ 30), based on World Health Organization (WHO) classification^7^] and the linear and quadratic terms for age were replaced by a binary indicator equalling one for those above the median age (39 years of age). The interactions of these two variables were also added to the model.

## Results

Supplementary appendix table A2 reports the estimated odds ratios (ORs), their 95% confidence intervals (CIs) and significance levels for both sexes in Model 1. The parameter estimates for the BMI b-spline components were omitted from the table since they are not interpretable as such. The results showed that daily smoking and a higher number of hospital visits were associated with a higher likelihood of premature retirement. Furthermore, marriage and cohabitation were associated with a lower likelihood of premature retirement. For men, there were no significant differences in the risk of premature retirement between education levels, areas, or regions of residence, whereas for women, obtaining the highest education level significantly decreased the risk of premature retirement but residing in a rural or an urban region increased the risk.

Figure 1 illustrates the relationship between BMI and the probability of premature retirement with the 95% confidence bands as modelled by the b-spline for men and women. For the predictions shown in the figure, the age of the person was set to the Health 2000 participants’ median age (i.e. 39 years of age). Furthermore, the categorical covariates were set to their reference values, indicating that these predictions apply to a person from a high population density urban region in the HYKS university hospital district, with the lowest education level, who had zero hospital visits during the past 5 years, never smoked and was not married or cohabiting. Panel A indicates a J-shaped relationship between BMI and the probability of premature retirement for men. Additionally, there was a BMI threshold value, deviations from which increased the risk of premature retirement. This BMI threshold value was approximately 23 for men. Compared to the threshold value, the likelihood of premature retirement for men was 1.9, 1.1, 1.5 and 1.8 times as high at BMI = 20, 25, 30 and 35, respectively. Instead of finding a similar J-shaped relationship in Panel B for women, we observed an almost linear relationship between BMI and the probability of premature retirement. Compared to a BMI of 20, the likelihood of premature retirement for women was 1.1, 1.4 and 1.6 times as high at BMI = 25, 30 and 35, respectively. It should be noted that there was greater uncertainty in the predictions for men, as indicated by the wider confidence band at the extreme values of BMI. However, both b-splines still significantly differed from zero (men: *P* < 0.05; women: *P* < 0.01).

Supplementary appendix table A3 reports the estimated ORs, their 95% CIs and significance levels for both sexes in Model 2, where WC instead of BMI was used as the predictor. The results were very similar to the ones obtained using Model 1. Significant point estimates were observed in the same predictors as in Model 1, with some slight numerical differences.

**Figure 2:**
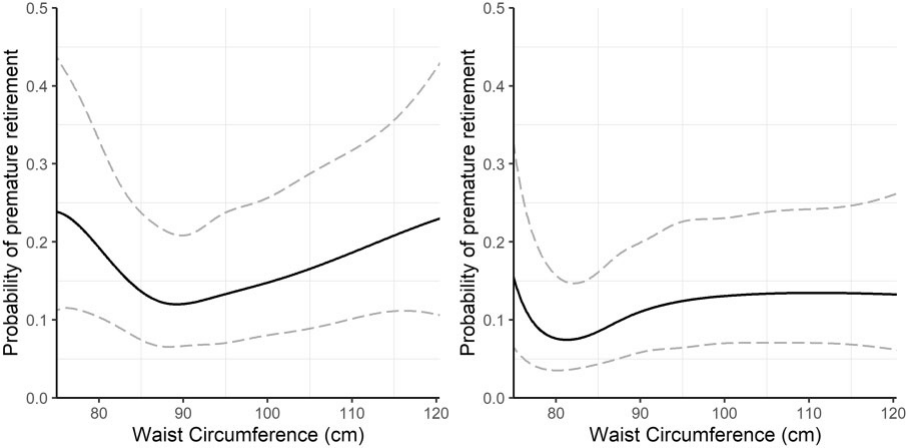
The relationship between WC and the probability of premature retirement was modelled by b-splines with six degrees of freedom. For the predictions, the background variables were set as follows: 39 years of age, high density area of residence, HYKS university hospital district, primary level of education, zero hospital visits during the past 5 years, never smoked, not married or cohabiting. Note: *N* = 1167 (men), 1318 (women)

Figure 2 illustrates the relationship between WC and the probability of premature retirement, with their 95% confidence bands as modelled by the b-spline for men and women. The covariate values in the predictions were set identically as in the case of Model 1. Panel A indicates a J-shaped relationship between WC and the probability of premature retirement for men, similar to that of figure 1. For women, the relationship between WC and premature retirement in Panel B was also similar to that of the J-shape observed in Panel A for men, but the effect was less pronounced compared to men. Thus, there appeared to be a WC threshold value, where the predicted probability of premature retirement was the smallest (slightly below 90 cm for men and slightly above 80 cm for women). Compared to the threshold value, the likelihood of premature retirement for men was 1.6, 1.2 and 1.9 times as high at WC = 80, 100, and 120, respectively. Similarly for women, the likelihood was 2.1, 1.5 and 1.8 times as high when compared to the threshold value at WC = 75, 90 and 115, respectively. However, in this case, the b-splines were not significant (men: *P* > 0.30, women: *P* > 0.13).

In models without the number of hospital visits, the parameter estimates and the overall shape of the relationship did not change notably compared to the full models for either BMI or WC (see Supplementary appendix tables A4 and A5 and figures A2 and A3). The second robustness test, where continuous BMI and age were replaced with indicator variables and their interactions, showed that none of the interaction terms were statistically significant (see Supplementary appendix table A6).

## Discussion

The increasing prevalence of obesity has been one of the most striking global health trends during the past few decades. Besides direct medical costs to the healthcare system, overweight and obesity are accompanied by significant indirect costs that result from productivity and employment losses in the labour market. It has been estimated that these indirect costs of obesity are comparable to or may even exceed the direct medical costs related to obesity [e.g. Goettler et al.^22^]. The productivity losses in the labour market result from lower productivity while working, sickness absenteeism, unemployment and premature retirement.

Using longitudinal data that combined medical examination data with survey and register data on prime working-age individuals, we found that high body weight was associated with an increased risk of premature retirement for both men and women. This result is consistent with previous studies, which found that excess weight increases the risk of premature exit from the labour force through the disability pension.^8^^,^^9^ When examining the association between BMI and the probability of premature retirement, we discovered a J-shaped relationship for men. Thus, at low BMI levels, an increase in BMI was linked with a lower risk of premature retirement, but after a threshold point (at approximately BMI = 23), the relationship was reversed. A related finding has been made using Swedish data, where a J-shaped relationship between BMI and the incidence of disability pension was reported for men who underwent military conscription induction tests.^11^^,^^12^^,^^23^ We did not find a J-shaped relationship for women, for whom the probability of premature retirement increased almost linearly with BMI. When WC was used instead, we observed similar coefficients in the model covariates, but the relationship between WC and the probability of premature retirement was slightly different from that of BMI. While the relationship between BMI and premature retirement for women was linear, the link was J-shaped for the case of WC. For men, the relationship also remained J-shaped when WC was used as an indicator of body mass. The BMI and WC threshold levels were close to the WHO cut-off points for excess weight.^7^^,^^24^ The most notable differences in the probability of premature retirement between women and men were at the lower end of the BMI distribution. The finding that a very low body mass seemed to be more detrimental for men than women may be linked to occupational segregation in terms of physical demands of occupations or body-composition differences^25^ between sexes.

Socioeconomic status may also explain the relationship between obesity and premature retirement. Typically, more educated individuals have a lower BMI than their less-educated counterparts.^26^^,^^27^ Moreover, highly educated individuals often work in less physically demanding occupations, retire later, and are less likely to enter the disability pension.^28^ As education may explain the relationship between excess weight and premature retirement, we used educational attainment as a control in all our models. We also adjusted for the number of hospital visits, marital status and smoking behaviours. Although socioeconomic status, marital status, smoking behaviour and hospital visits may partially account for the association between excess weight and premature retirement, they did not fully explain this relationship.

Our empirical approach has limitations that must be noted when causal interpretations are considered. First, survey data are prone to non-participation, and the Health 2000 Survey is not an exception in this regard. However, the participation rate in the Health 2000 health examinations was very high (84%). Furthermore, we used inverse probability weights^17^ to correct the effects of non-participation and provide nationally representative results. Second, although we controlled for several potential confounders (such as education and area of residence), we cannot rule out the possibility that unobserved heterogeneity may affect our results. Third, we did not examine potential mediators that may explain the link between BMI and premature retirement in this study. Obesity has been linked to weaker health status and chronic obesity-related illnesses (such as type-2 diabetes, high blood pressure, high cholesterol and atherosclerosis) in the medical literature.^29^ Thus, it is possible that poor health at least partially mediates the link between excess weight and early retirement. This notion has received support from studies that found that individuals who suffer from obesity are more likely to take sick leave.^30–32^ The main reasons for receiving the disability pension in Finland are mental disorders (52%) and musculoskeletal diseases (19%).^19^ As obesity has been identified as a major risk factor for mental disorders [e.g. Scott et al.^33^] and musculoskeletal diseases [e.g. Anandacoomarasamy et al.^34^], it is possible that excess weight partially explains exits from the labour market through disability pension. Future studies could shed further light on mediators that may explain the link between excess weight and premature retirement. Fourth, for a person to be entitled to disability-related pension, the health problems have to be relatively severe. Thus, individuals who are not entitled to disability pension may try to find other ways to cope with their health problems in the labour market. Previous studies have shown that obesity is associated with a lower employment probability [e.g. Böckerman et al.^35^] and a higher unemployment risk [e.g. Bramming et al.^36^]. Whether these other potential routes of employment exit consist an alternative to premature retirement is, however, beyond the scope of this paper.

The main strength of this study comes from the representative population-based survey^16^^,^^37^ that was linked to register information on actual retirement ages. Weight, height and WC were based on objective measures obtained by trained nurses using standardized methods. Furthermore, information on premature retirement was obtained from register-based data, which eliminated the potential bias of self-reported measures.

In conclusion, this Finnish nationally representative study showed that excess weight and increased WC were associated with a higher probability of premature retirement. The results suggest that the promotion of a healthy lifestyle and obesity prevention may decrease the risk of premature exit from the labour market. Further research, however, is needed to confirm the causal interpretation of the association. Our findings were based on data from Finland, where access to health care is universal. Although everyone residing in Finland is entitled to public health care, we found a large and robust link between excess weight and premature retirement. In countries where such a healthcare system does not exist, the link between excess weight and premature retirement may be even more pronounced.

## Supplementary data

Supplementary data are available at *EURPUB* online.

## Funding

S.T. was funded by Academy of Finland (grant number 311877).

*Conflicts of interest:* None declared. 

## Data availability

The data underlying this article cannot be shared publicly due to the privacy of individuals. Researchers can request access to the Health 2000 data from the THL Biobank.

**Key points**

- Health status is a principal determinant of long-term labour market participation.
- Excess body weight is associated with premature retirement in Finland.
- The association varies between sexes and across the weight distribution.
- Body weight outside a range recommended by WHO increases the risk of premature retirement.

## Supplementary Material

ckab116_Supplementary_Data
