# Supplementary material for: Body weight and premature retirement: population-based evidence from Finland
Source: Eur J Public Health. 2021 Jul 19;31(4):731–6. doi: 10.1093/eurpub/ckab116 (PMC8514174; doi:10.1093/eurpub/ckab116)
Supplement: ckab116_Supplementary_Data [file ckab116_supplementary_data.docx]

**Appendix**

Appendix Figure 1. Flowchart of the Health 2000 Survey and the construction of the study sample.


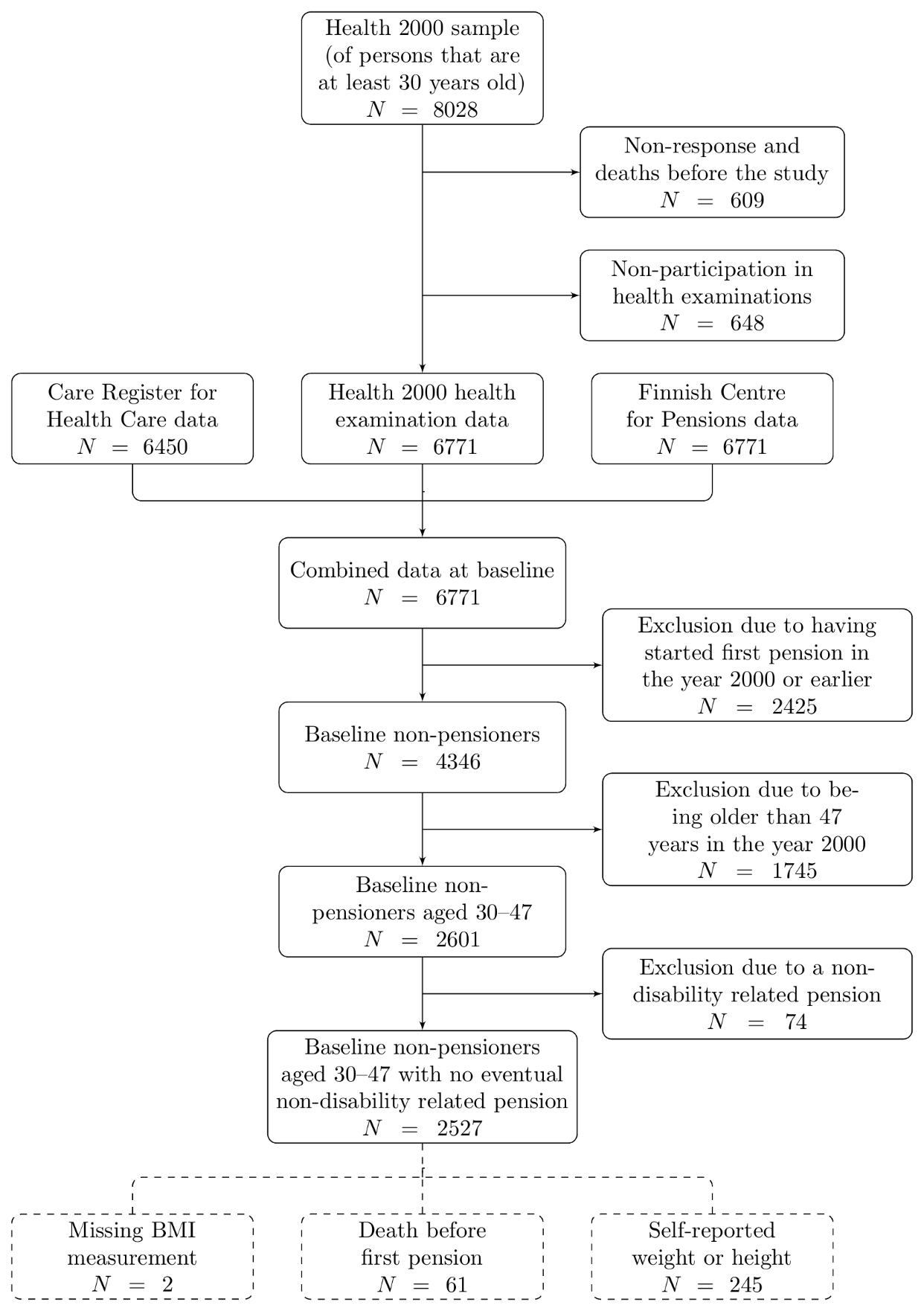


Appendix Table 1. Descriptive statistics of the study sample by BMI category.

|  | Underweight  (BMI < 18.5) | Normal weight  (18.5 ≤ BMI < 25) | Overweight  (25 ≤ BMI < 30) | Obese  (BMI ≥ 30) |
| --- | --- | --- | --- | --- |
| Median age | 38 | 38 | 39 | 40 |
| Men | 5 (20.0%) | 466 (38.7%) | 524 (57.5%) | 187 (48.8%) |
| Women | 20 (80.0%) | 739 (61.3%) | 388 (42.8%) | 196 (51.1%) |
| Premature  retirement | 9 (36%) | 148 (12.3%) | 135 (14.8%) | 74 (19.3%) |
| Observations | 25 | 1205 | 912 | 383 |

**Notes to Appendix Tables 2–5**: The outcome variable equalled one for those who retired or died before the age of 63 over the period 2001–2015 and zero otherwise. BMI (WC) was included in the model as a cubic b-spline function with six degrees of freedom. The spline was constructed separately for both sexes by placing knots at the lower quartile, median, and upper quartile of the empirical BMI (WC) distributions of the sexes. The reference groups were as follows: Smoking: non-smoked; education: primary education; area: HYKS; region: high density urban; marital status: not married or cohabiting; number of hospital visits: none. Abbreviations: BMI = body mass index, WC = waist circumference, HYKS = Helsinki University Hospital district, TYKS = Turku University Hospital district, TaYS = Tampere University Hospital district, KYS = Kuopio University Hospital district, OYS = Oulu University Hospital district.

Appendix Table 2. Results from logistic regression using BMI (Model 1).

|  | Men | Women |
| --- | --- | --- |
| Age | 0.756 (0.415, 1.379) | 0.614 (0.350, 1.076) |
| Age^2^ | 1.005 (0.997, 1.013) | 1.008 (1.001, 1.015) |
| Smoking: occasionally | 0.756 (0.301, 1.330) | 1.750 (0.987, 3.103) |
| Smoking: daily | 1.924** (1.306, 2.835) | 1.829** (1.247, 2.682) |
| Education: middle | 0.794 (0.513, 1.229) | 0.946 (0.608, 1.473) |
| Education: highest | 0.642 (0.372, 1.106) | 0.480** (0.299, 0.771) |
| Area: TYKS | 1.120 (0.643, 1.949) | 1.276 (0.758, 2.149) |
| Area: TaYS | 0.706 (0.412, 1.209) | 0.747 (0.461, 1.210) |
| Area: KYS | 1.172 (0.673, 2044) | 0.898 (0.531, 1.520) |
| Area: OYS | 1.210 (0.680, 2.154) | 1.277 (0.765, 2.133) |
| Region: low density urban | 1.346 (0.790, 2.295) | 1.737* (1.035, 2.913) |
| Region: rural | 1.035 (0.647, 1.656) | 1.558* (1.038, 2.337) |
| Married or cohabiting | 0.481** (0.330, 0.701) | 0.640* (0.443, 0.927) |
| Hospital visits: 1–3 | 1.222 (0.787, 1.898) | 1.140 (0.780, 1.666) |
| Hospital visits: >3 | 3.188** (1.835, 5.538) | 3.930** (2.529, 6.106) |
| Observations | 1176 | 1336 |

Note: The results were based on a logistic regression model. The table presents odds ratios and their 95% confidence intervals in parentheses. The parameter estimates for the BMI b-spline components were omitted from the table since they are not interpretable as such. *p < 0.05; **p < 0.01.

Appendix Table 3. Results from logistic regression using WC (Model 2).

|  | Men | Women |
| --- | --- | --- |
| Age | 0.804 (0.442, 1.464) | 0.647 (0.365, 1.147) |
| Age^2^ | 1.004 (0.997, 1.012) | 1.007 (1.000, 1.015) |
| Smoking: occasionally | 0.632 (0.229, 1.335) | 1.747 (0.978, 3.122) |
| Smoking: daily | 1.946** (1.311, 2.887) | 1.857** (1.256, 2.745) |
| Education: middle | 0.812 (0.521, 1.266) | 0.927 (0.593, 1.449) |
| Education: highest | 0.647 (0.374, 1.119) | 0.478** (0.297, 0.770) |
| Area: TYKS | 1.126 (0.644, 1.969) | 1.251 (0.738, 2.123) |
| Area: TaYS | 0.718 (0.421, 1.224) | 0.724 (0.444, 1.181) |
| Area: KYS | 1.177 (0.672, 2.062) | 0.832 (0.487, 1.420) |
| Area: OYS | 1.131 (0.630, 2.032) | 1.169 (0.695, 1.965) |
| Region: low density urban | 1.328 (0.773, 2.283) | 1.735* (1.032, 2.917) |
| Region: rural | 1.077 (0.672, 1.724) | 1.534* (1.014, 2.320) |
| Married or cohabiting | 0.483** (0.330, 0.706) | 0.646* (0.446, 0.937) |
| Hospital visits: 1–3 | 1.210 (0.778, 1.883) | 1.165 (0.795, 1.707) |
| Hospital visits: >3 | 3.175** (1.816, 5.551) | 4.041** (2.597, 6.287) |
| Observations | 1167 | 1318 |

Note: The results were based on a logistic regression model. The table presents odds ratios and their 95% confidence intervals in parentheses. The parameter estimates for the WC b-spline components were omitted from the table since they are not interpretable as such. *p < 0.05; **p < 0.01.

Appendix Table 4. Results from logistic regression using BMI without hospital visits (Model 3).

|  | Men | Women |
| --- | --- | --- |
| Age | 0.783 (0.432, 1.419) | 0.651 (0.372, 1.138) |
| Age^2^ | 1.005 (0.997, 1.012) | 1.007 (1.000, 1.014) |
| Smoking: occasionally | 0.635 (0.302, 1.337) | 1.786* (1.007, 3.167) |
| Smoking: daily | 1.924** (1.317, 2.811) | 1.739** (1.194, 2.532) |
| Education: middle | 0.758 (0.490, 1.174) | 1.016 (0.655, 1.578) |
| Education: highest | 0.629 (0.365, 1.082) | 0.538** (0.336, 0.859) |
| Area: TYKS | 1.084 (0.623, 1.888) | 1.263 (0.763, 2.090) |
| Area: TaYS | 0.705 (0.417, 1.192) | 0.735 (0.458, 1.181) |
| Area: KYS | 1.189 (0.684, 2066) | 0.822 (0.490, 1.381) |
| Area: OYS | 1.177 (0.660, 2.099) | 1.204 (0.732, 1.983) |
| Region: low density urban | 1.326 (0.784, 2.237) | 1.636 (0.979, 2.735) |
| Region: rural | 0.973 (0.609, 1.555) | 1.538* (1.038, 2.278) |
| Married or cohabiting | 0.486** (0.334, 0.705) | 0.674* (0.471, 0.965) |
| Observations | 1176 | 1336 |

Note: The results were based on a logistic regression model. The table presents odds ratios and their 95% confidence intervals in parentheses. The parameter estimates for the BMI b-spline components were omitted from the table since they are not interpretable as such. *p < 0.05; **p < 0.01.

Appendix Table 5. Results from logistic regression using WC without hospital visits (Model 4).

|  | Men | Women |
| --- | --- | --- |
| Age | 0.832 (0.460, 1.506) | 0.680 (0.384, 1.203) |
| Age^2^ | 1.004 (0.996, 1.011) | 1.007 (0.999, 1.014) |
| Smoking: occasionally | 0.635 (0.301, 1.340) | 1.785 (0.999, 3.188) |
| Smoking: daily | 1.951** (1.326, 2.871) | 1.755** (1.197, 2.574) |
| Education: middle | 0.776 (0.499, 1.207) | 0.983 (0.631, 1.531) |
| Education: highest | 0.634 (0.368, 1.094) | 0.533** (0.333, 0.852) |
| Area: TYKS | 1.089 (0.625, 1.896) | 1.226 (0.737, 2.037) |
| Area: TaYS | 0.718 (0.425, 1.211) | 0.707 (0.437, 1.144) |
| Area: KYS | 1.201 (0.686, 2.103) | 0.753 (0.444, 1.277) |
| Area: OYS | 1.104 (0.613, 1.990) | 1.101 (0.667, 1.819) |
| Region: low density urban | 1.309 (0.770, 2.228) | 1.624 (0.970, 2.718) |
| Region: rural | 1.013 (0.635, 1.617) | 1.522* (1.019, 2.271) |
| Married or cohabiting | 0.488** (0.336, 0.710) | 0.679* (0.473, 0.934) |
| Observations | 1167 | 1318 |

Note: The results were based on a logistic regression model. The table presents odds ratios and their 95% confidence intervals in parentheses. The parameter estimates for the WC b-spline components were omitted from the table since they are not interpretable as such. *p < 0.05; **p < 0.01.

Appendix Table 6. Results from logistic regression using categorical BMI, age and their interaction.

|  | Men | Women |
| --- | --- | --- |
| Age | 2.341 (0.139, 39.48) | 3.730 (0.493, 23.46) |
| Normal weight | 0.185 (0.024, 1.398) | 0.316 (0.075, 1.335) |
| Overweight | 0.255 (0.034, 1.924) | 0.443 (0.103, 1.913) |
| Obese | 0.362 (0.046, 2.854) | 0.452 (0.094, 2.164) |
| Age: Normal weight | 1.351 (0.075, 24.23) | 0.740 (0.111, 4.947) |
| Age: Overweight | 1.020 (0.058, 18.09) | 0.659 (0.096, 4.509) |
| Age: Obese | 0.962 (0.052, 17.78) | 0.802 (0.107, 6.030) |
| Observations | 1176 | 1336 |

Note: The results were based on a logistic regression model. The table presents odds ratios and their 95% confidence intervals in parentheses. The age parameter equalled one if the participant’s age was above the median age (39 years). The reference weight was underweight (BMI < 18.5). Other covariates were the same as in Model 1, but their parameter estimates were omitted from this table for brevity. *p < 0.05; **p < 0.01.

Appendix Figure 2. Predicted probability of premature retirement for the reference individual and its 95% confidence band as a function of BMI in the model without hospital visits (Model 3).

Panel A: Men Panel B: Women


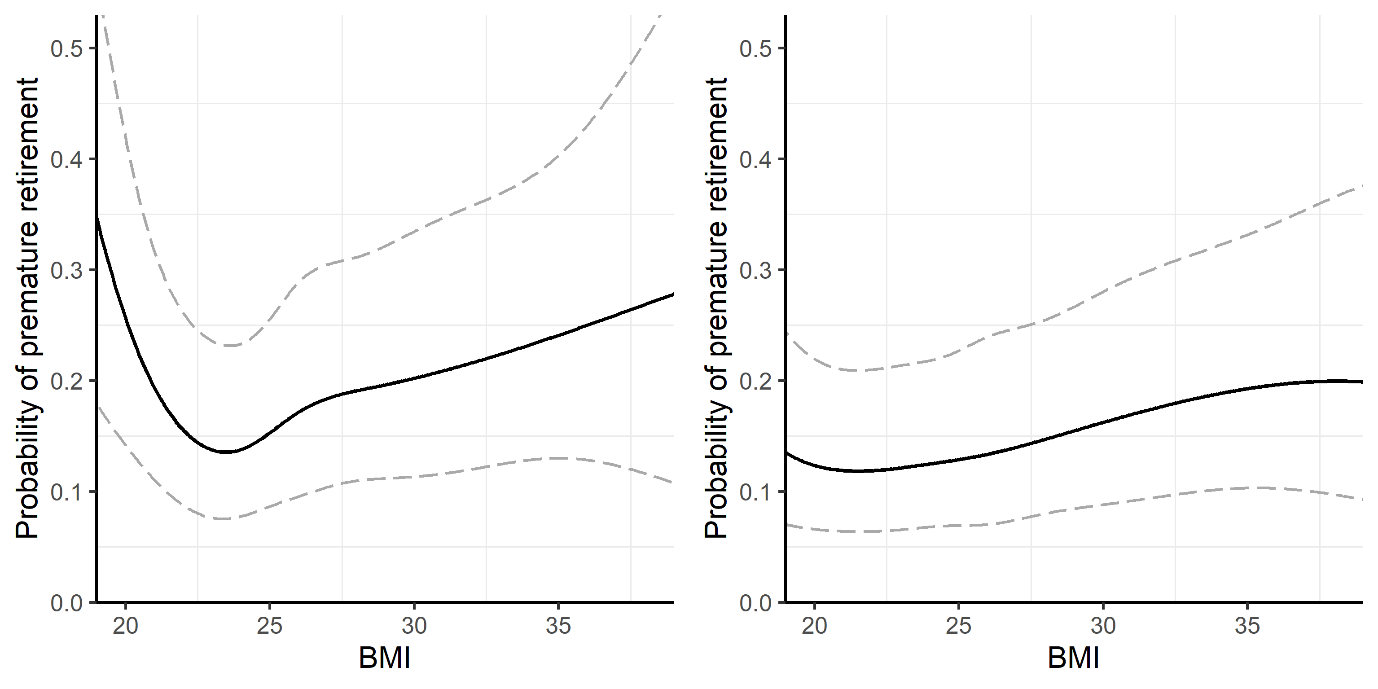


Note: N = 1176 (men), 1336 (women). The relationship between BMI and the probability of premature retirement was modelled by b-splines with six degrees of freedom. For the predictions, the background variables were set as follows: 39 years of age, high density area of residence, HYKS university hospital district, primary level of education, zero hospital visits during the past five years, never smoked, not married or cohabiting.

Appendix Figure 3. Predicted probability of premature retirement for the reference individual and its 95% confidence band as a function of WC in the model without hospital visits (Model 4).

Panel A: Men Panel B: Women


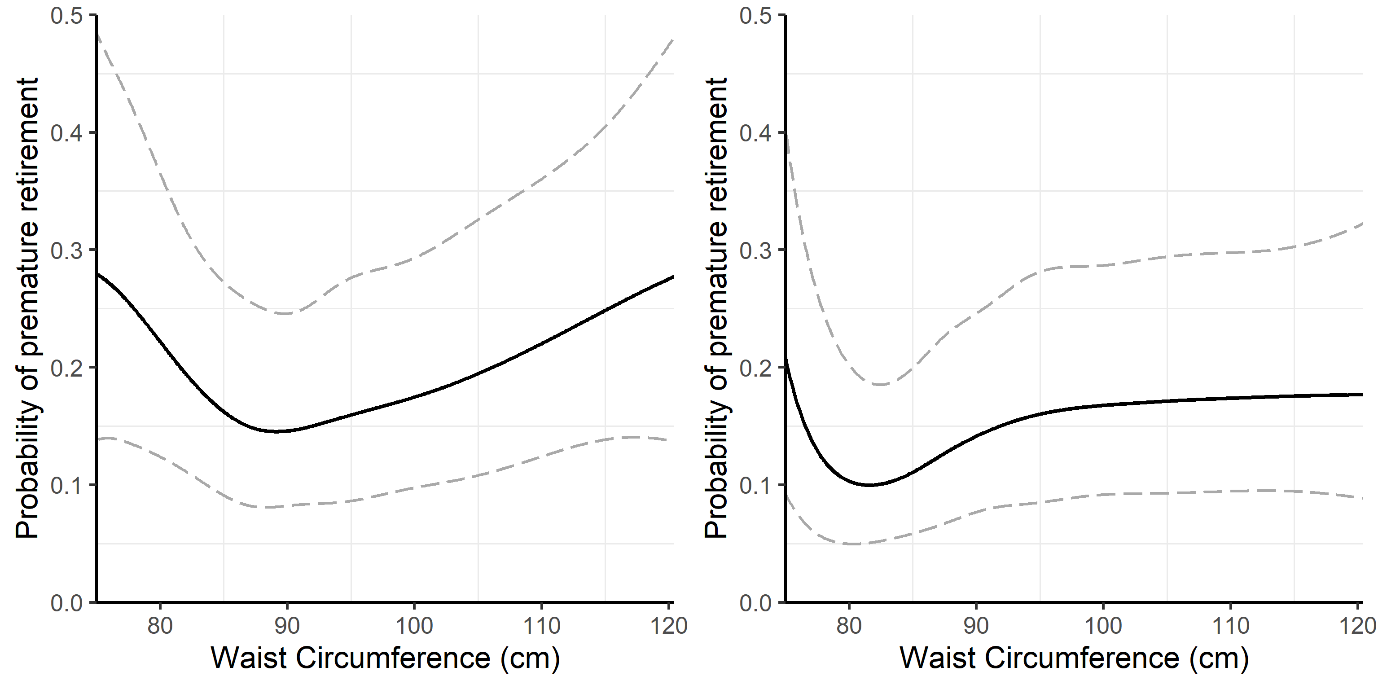


Note: N = 1167 (men), 1318 (women). The relationship between WC and the probability of premature retirement was modelled by b-splines with six degrees of freedom. For the predictions, the background variables were set as follows: 39 years of age, high density area of residence, HYKS university hospital district, primary level of education, zero hospital visits during the past five years, never smoked, not married or cohabiting.
